# Genome-wide identification and expression analysis of YTH domain-containing RNA-binding protein family in maize reveals their potential role under abiotic stresses

**DOI:** 10.1038/s41598-026-53179-y

**Published:** 2026-05-18

**Authors:** Hameed Gul, Shareef Gul, Gao Jing, Rakia Manzoor, Muhammad Mudasir, Liu Qiuyi, Sumit Singh, Ali Shahzad

**Affiliations:** 1 College of Agronomy and Biotechnology, Southwest University, Beibei, Chongqing, 400715 China; 2 School of Economic and Management, Southwest University, Beibei, Chongqing, 400715 China; 3 Engineering Research Center of Coptis Development and Utilization (Ministry of Education), College of Pharmaceutical Sciences, Southwest University, Chongqing, 400715 China; 4 Department of Crop Sciences and Agroforestry, Faculty of Tropical AgriSciences, Czech University of Life Sciences Prague, Kamýcká 129, 16500 Prague 6, Czech Republic; 5 Department of Biological Sciences, Hunan Agricultural University, Changsha, 410128 China; 6 Department of Zoology, Guru Nanak Dev University, Amritsar, 143005 Punjab India; 7 School of Tropical Agriculture and Forestry, Hainan University, Danzhou, 571737 China; 8 School of Breeding and Multiplication (Sanya Institute of Breeding and Multiplication), Hainan University, Sanya, 572025 Hainan China

**Keywords:** YTH domain proteins, YTH gene family, m^6^A RNA modification, *Zea mays*, Genome-wide identification, Abiotic stress response, Biotechnology, Computational biology and bioinformatics, Genetics, Molecular biology, Plant sciences

## Abstract

Post-transcriptional control of gene expression is one of the strategies in plants to regulate growth, development, and stress adaptation. YT521-B homology (YTH) domain-containing RNA-binding proteins are essential for this post-transcriptional control through influencing the fate of RNA molecules containing N6-methyladenosine (m^6^A). Here, we conducted a genome-wide identification and characterization analysis of the YTH gene family in maize (*Zea mays* L.) and found 22 *ZmYTHs*. Phylogenetic analysis showed that the ZmYTH could be divided into four groups, while the *ZmYTH* genes are distributed across ten chromosomes. Subcellular localization analysis suggests that most ZmYTH proteins often exhibit a nuclear localization consistent with their involvement in RNA metabolism. Duplication of genes, in particular segmental and whole-genome duplications, has driven the proliferation of this gene family, potentially endowing maize with greater capacity to regulate stress-responsive pathways. Further, we identified 31 cis-regulatory elements in the promoter regions of *ZmYTH* genes involved in development, stress, and hormonal regulation. Protein-protein interaction prediction and miRNA target prediction further elaborate their role in post-transcriptional modification. We profiled *ZmYTH’s* expression pattern across development and stress conditions, confirming 10 genes differentially expressed under drought, salt, and heat stresses. *ZmYTH1* and *ZmYTH3* were consistently induced, *ZmYTH7* was repressed under all three conditions, and the remaining genes showed condition-specific patterns. Overall, these findings highlight the key role of the *ZmYTH* family in stress adaptation and provide a basis for future studies on its molecular mechanisms.

**Supplementary Information:**

The online version contains supplementary material available at 10.1038/s41598-026-53179-y.

## Introduction

 The post-transcriptional regulation of gene expression is important for plant growth and development, as well as for adaptation to environmental stress^1,2^. The processes involved in this regulatory layer are pre-mRNA processing, polyadenylation, mRNA stability, translation, and RNA transport^3^. Although the mediating role of the epigenetic alterations (e.g., DNA methylation and histone modifications) in transcriptional regulation is a major part of the gene expression regulation^4^, post-transcriptional chemical modification of RNA molecules can also be affected by various chemical changes that affect their stability, processing, and functionality^5,6^. The most common of these modifications is the N6-methyladenosine (m^6^A) internal modification that occurs in eukaryotic mRNA^7^. Such a change is fundamental to the regulation of RNA splicing, transcript stability, translation efficiency, and degradation^8^. Since m^6^A is an evolutionarily conserved regulatory tag, it is involved in normal plant development and in dynamic responses to environmental cues. Some aspects of m^6^A modification that have been demonstrated in plants include cellular homeostasis and adaptive reaction to abiotic stresses, including drought, heat, and salinity^9,10^.

The dynamic regulation of m^6^A modification comprises three categories of proteins: writers (methyltransferases), erasers (demethylases), and readers^11^. The process of methylating specific adenosine residues is catalyzed by the writer complex comprising METTL3, METTL14, WTAP, and RBM15/RBM15B^12^. Such methylation patterns typically occur at conserved RRACH consensus motifs (R = A/G; H = A/C/U). These methyl groups are removed by demethylases like FTO and ALKBH5^13^, allowing a reversible and dynamic control of mRNA methylation. Nevertheless, the biological consequences of m^6^A modification are mainly mediated by reader proteins, which selectively identify methylated transcripts and regulate downstream events. These m6A readers are primarily RNA-binding proteins (RBPs) that regulate the stability of transcripts, their ability to be translated, their localization, and alternative splicing^13,14^.

Early mechanistic studies of the m^6^A regulation were mostly based on animal and yeast models^15^, where this modification was found to regulate mRNA transport, translation and stability^16^. Although m^6^A research in plants emerged later and initially lagged behind animal studies, the adoption of techniques from other model systems has significantly advanced the field^17^. Consequently, m6A modification has become a significant focus in plant biology, especially for understanding regulatory responses to stress, growth, and development^18^. One of these m^6^A reader proteins, the YT521-B homology (YTH) domain, is a canonical m^6^A-binding factor that converts methylation cues into specific regulatory outcomes, and it is a key participant in post-transcriptional gene regulation in plants^11,19^.

Research on RNA-binding proteins (RBPs) in plants was originally constrained by the lack of appropriate in vitro models to study post-transcriptional gene regulation^17,20^. Nevertheless, molecular tools and technologies have significantly shortened the time of the identification and functional characterization of plant RBPs^21^. The YT521-B homology (YTH) domain was first discovered in 2002 by Stoilov et al. and is a conserved RNA-binding domain found in nuclear proteins^22^. The YTH domain typically contains 100–150 amino acids and is highly conserved in eukaryotes. It has a conserved aromatic cage that specifically binds to m^6^A-modified adenosine residues, allowing YTH proteins to interact with methylated transcripts to regulate RNA stability, translation, and alternative splicing^19,23^. The functional specialization and diversification of post-transcriptional regulation in plants imply that the expansion of YTH domain-containing proteins has undergone lineage-specific diversification. A number of YTH proteins have been found in other species, including *Arabidopsis thaliana* and *Oryza sativa*, since their discovery^24,25^. The proteins play roles in regulating plant-specific processes, such as hormone signaling and stress responses^26^.

For instance, the high expression of apical meristem and young leaves in Arabidopsis includes the methyltransferases MTA and FIP37 (orthologs of METTL3 and WTAP), which have been shown to regulate cell proliferation and embryogenic processes^27^. Besides, the Arabidopsis demethylase ALKBH10b removes m^6^A marks from flowering-time gene transcripts, including FT, SPL3, and SPL9, thereby controlling the floral transition^28^. Stress adaptation and developmental regulation of m^6^A modification also play roles in fruit development. For example, MTA and MTB regulate transcripts involved in the ABA biosynthesis and signaling pathway (NCED5, AREB1, and ABAR) during strawberry fruit ripening^29^. Similarly, MdMTA improves m^6^A modification of stress-responsive genes in apple under drought conditions, thereby increasing mRNA stability and translation efficiency^30^. This is facilitated gene expression that facilitates drought tolerance by depositing lignin and lysing reactive oxygen species. All of these studies emphasize the significant functions of m^6^A modification in plant stress responses and development^10^. More recent studies have also shown that YTH proteins are major mediators of m^6^A regulation in plants^23^. Five YTH proteins have been discovered in humans (YTHDC1-2 and YTHDF1-3); YTHDC1 and YTHDC2 are nuclear m6A readers, and YTHDF1-3 perform their functions primarily in the cytoplasm^19^. Next-generation sequencing technologies have rapidly advanced, and genome-wide identification and evolutionary analysis of YTH proteins have been reported in various plant species, including Arabidopsis, rice^30^, tomato^31^, alfalfa^32^, and wheat^31^. The expression of the YTH genes in Arabidopsis is associated with responses to abiotic stresses, including cold, drought, salinity, and heat, further confirming their functions in stress tolerance^32^.

Maize (*Zea mays* L.) is one of the most important cereal crops worldwide and represents a major source of food, feed, and biofuel production^33,34^. However, maize productivity is usually constrained by abiotic stresses, including drought, salinity, and heat, which significantly affect plant growth and yield stability^35^. Although m6A-based regulation and YTH domain-containing proteins have been described in model plants, including Arabidopsis and rice^23,24^, the YTH gene family in maize has not been extensively studied. Considering the key role of YTH proteins in interpreting m^6^A signals and regulating RNA metabolism, a comprehensive analysis of maize YTH genes is necessary to better understand post-transcriptional regulatory processes in stress adaptation and to serve as the basis for future functional studies and crop enhancement strategies. Thus, the objectives of the study were to genome-wide identify and characterize YTH domain-containing genes in maize and to examine their evolutionary lineages, structural attributes, and expression patterns.

## Materials and methods

### Identification of the YTH proteins

The genome database of *Sorghum bicolor* (Sorghum_bicolor_NCBIv3), *O. sativa* Japonica Group (IRGSP-1.0), *Triticum aestivum* (IWGSC), and *Z. mays* (Zm-B73-REFERENCE-NAM 5.0) were retrieved from the Ensembl Plants website (http://plants.ensembl.org/index.html). Conversely, the genome database of *A. thaliana* (TAIR10) was downloaded from the TAIR website (http://www.arabidopsis.org). Similarity-based and profile-based search strategies were used to achieve the accurate and comprehensive identification of YTH domain-containing proteins in maize. The 13 Arabidopsis YTH protein sequences were used as queries for local BLASTp searches with an E-value threshold of 1e^−5^. This cutoff was chosen to reduce false-positive matches and retain possibly divergent homologs. Besides that, an alignment coverage of 50% was used as a minimum requirement^36–38^ to ensure that candidate sequences had significant similarity to known YTH proteins and to remove short or fragmented sequences lacking conserved structural motifs.

Simultaneously, the Hidden Markov Model (HMM) profile of the conserved YTH domain (PF04146) was accessed at Pfam database and searched on HMMER to find sequences that contain the characteristic YTH domain. A combination of BLASTp and HMM-based searches increased detection sensitivity and reduced the risk of overlooking divergent family members. Candidate sequences were further validated using Pfam (https://www.ebi.ac.uk/Tools/pfa/pfamscan/, Table S1), SMART (https://smart.embl.de/), and the NCBI Conserved Domain Database (CDD) (http://www.ncbi.nlm.nih.gov/Structure/cdd/wrpsb.cgi) to confirm the presence of intact YTH domains. Lastly, redundant sequences and alternative transcript forms were filtered by gene ID, and only non-redundant protein sequences with a full YTH domain were used in the analyses.

### Prediction of ZmYTH proteins physicochemical characteristics and subcellular localization

The physicochemical properties of the ZmYTH proteins were predicted using ProtParam (http://web.expasy.org/protparam/) from the ExPASy server. The parameters calculated included amino acid composition, molecular weight (MW), isoelectric point (pI), and Grand Average of Hydropathy (GRAVY). Subcellular localization of the ZmYTH proteins was predicted using the Plant-mPLoc program (http://www.csbio.sjtu.edu.cn/bioinf/plant-multi), as described by^36^.

### Phylogenetic analysis, gene structure, domains, and conserved motif analysis

To examine the evolutionary connections between YTH proteins in *Z. mays*, *S. bicolor*, *O. sativa*, *A. thaliana*, and *T. aestivum*, the protein sequences were initially aligned with ClustalW. A phylogenetic tree was subsequently constructed using MEGA11 ^37^ based on the Neighbor-Joining (NJ) method with the JTT + I+G substitution model. Bootstrap analysis with 1000 replicates was performed to evaluate the reliability of the inferred phylogenetic relationships. The resulting phylogenetic tree was visualized using iTOL^38^. Both the exon-intron patterns of the *ZmYTH* genes were visualized with the help of GSDS 2.0 (http://gsds.gao-lab.org) and TBtools^39^. Meanwhile, MEME V5.5.3 (https://meme-suite.org/meme/) was used to predict conserved motifs using the following parameters: motif width of 6-100 residues, a maximum of 15 motifs, and an E-value threshold of ≤ 1 × 10^−10^.

### Chromosomal location, gene duplication, and synteny analysis

The chromosomal positions of the *ZmYTH* genes were obtained from the maize genome annotation file and visualized using MapChart v2.32^40^. Gene duplication events were detected with MCScanX, and synteny analysis was performed with TBtools using the Multiple Synteny Plotter. The synteny relationships between orthologous *ZmYTHs* were visualized using TBtools-Amazing Super Circos.

### Evaluation of cis-acting elements in *ZmYTH* gene promoters

Promoter regions of the *ZmYTH* genes (2,000-bp upstream of the ATG start codon) were extracted using TBtools and analyzed for cis-acting regulatory elements using the PlantCARE web tool (http://bioinformatics.psb.ugent.be/webtools/plantcare/html/)^41^. The presence of potential regulatory elements was visualized using TBtools.

### Protein–protein interaction (PPI) network and miRNA prediction of *ZmYTH* genes

To investigate the potential regulatory mechanisms of ZmYTH proteins, we first predicted their protein-protein interactions (PPIs) using the STRING database (https://string-db.org/cgi/input.pl). Only interactions with a confidence score ≥ 0.4 (medium confidence), which corresponds to the default STRING threshold, were retained to improve the reliability of the predicted interaction network. In addition, to explore the potential post-transcriptional regulation of *ZmYTH* genes by miRNAs, we used the psRNATarget tool (https://www.zhaolab.org/psRNATarget/) to predict miRNA-target interactions^42^. The PPI network and miRNAs were visualized using Cytoscape 3.9.1^43^. The interaction data were exported in TSV format and imported into Cytoscape for further network analysis and visualization. This combined approach enabled us to identify key regulatory relationships between ZmYTH proteins and miRNAs, providing insights into their potential roles in stress-response regulation and gene expression.

### Expression profile of *ZmYTHs* in different tissues and under various stresses

The *ZmYTH* gene expression in 20 maize tissues was retrieved from the Maize Genome Database (http://maizegdb.org) with the qTeller tool (https://qteller.maizegdb.org/genes_by_name_B73v5.php)^44^. The analyzed tissues included root, leaf, internode, ear primordium, embryo, and silk at different developmental stages. The expression data of *ZmYTH* under various biotic (*Colletotrichum graminicola*, *Fusarium graminearum*,* Cercospora Zeina*,* Sugarcane mosaic virus*) and abiotic (Cold, drought, heat, salt, waterlogging, nitrogen, ozone) conditions were also retrieved on the qTeller platform. The expression levels were normalized using the log2(FPKM + 1) method, and the resulting values were used to generate expression heatmaps with TBtools. The heat maps provide a clear visual representation of the differential expression patterns of *ZmYTH* genes across various tissues and stress treatments.

### Plant growth, stress treatments, and qRT-PCR analysis

To examine the expression of *ZmYTH* genes in response to stress conditions, seeds of the maize inbred line B73 were obtained from the College of Agronomy and Biotechnology, Southwest University, China. Seeds were sterilized with 70% ethanol for 5 min, then rinsed with sterile water. Subsequently, they were put on Petri dishes with wet filter paper and maintained at 25 °C under a 16 h light/8 h dark photoperiod inside a growth chamber for germination. After seed germination, the seedlings were subjected to stress induction by transferring them to hydroponic growth plates at the three-leaf stage. Seedlings were subjected to the following stress conditions: 20% PEG 6000 in Hoagland’s solution to induce drought stress, 150 mM NaCl solution for salt stress, and a temperature of 38 °C for heat stress^45^. The leaf samples were collected at control (CK), 5, and 10 h post-treatment for RNA extraction and further quantification using qRT-PCR^46^.

For RNA extraction, leaf samples were frozen in liquid nitrogen and stored at − 80 °C until further processing. A total of 200 mg of RNA was isolated using TRIzol Reagent (Invitrogen, Carlsbad, CA, USA), and the quality and concentration of RNA were measured using a Nanodrop 2000 spectrophotometer (Thermo Fisher Scientific, Wilmington, DE, USA). The first-strand cDNA synthesis was performed by the PrimeScript™ RT Reagent Kit (TaKaRa, Shiga, Japan) in the presence of Oligo(dT) primers. qRT-PCR was conducted in an ABI7500 Real-Time PCR System (Applied Biosystems, Foster City, CA, USA) using *ZmACTIN* as the control gene. Each reaction was carried out in three biological triplicates, and the level of expression was analyzed using the 2^^−ΔΔCT^ method^47,48^. Primer sequences for qRT-PCR are provided in Supplementary Table S2.

## Results

### Genome-wide identification and characterization of *ZmYTHs* in maize

To identify the YTH proteins in maize, we searched the protein database of maize with the HMM profile of the YTH structural domain and 13 *Arabidopsis* YTH Proteins, respectively. Further, we applied various tools such as PFAMSCAN, SMART and NCBI CDD to confirm the presence of YTH domain. We removed redundant sequences, and finally 22 YTH proteins were obtained and named ZmYTH1 to ZmYTH22 according to their chromosomal position (Table 1). Each gene contains 1 to 6 transcripts, including *ZmYTH1* and *ZmYTH5* having maximum of 6 transcripts, indicating that these genes may be functionally diversified through alternative splicing. The amino acid length of the encoded proteins differs substantially from one another and ranges from 119 (ZmYTH4) to 748 (ZmYTH8) amino acids. The predicted molecular weight (MW) of the 22 ZmYTHs ranged from 13412.17 (ZmYTH4) to 81823.7 (ZmYTH8) Da (dalton). The pI value of ZmYTH proteins varied from 5.54 to 9.34, and the highest isoelectric point was shown by *ZmYTH18*. The instability index was greater than 40 for most proteins, suggesting they would be unstable in vitro. The GRAVY (Grand Average of Hydropathy) value for all ZmYTH proteins was negative, and the index ranged from − 0.029 (*ZmYTH11*) to − 0.894 (*ZmYTH14*), denoting that all ZmYTH proteins are hydrophilic proteins. Computational tools were used to predict subcellular localization, suggesting that most of the ZmYTH proteins (18/22) are likely located in the nucleus, which is consistent with their proposed roles in RNA metabolism and gene expression regulation. Two proteins, ZmYTH4 and ZmYTH7, were localized in the cytoplasm, and one protein, ZmYTH19, was chloroplast-targeted.

**Table 1:** A summary of detailed characteristics of YTH gene family members in maize.

Gene name	Gene ID	Chromosome location	Number of transcripts	Length of amino acid (AA)	Molecular weight (MW)	pI	Instability index	GRAVY	Subcellular location
ZmYTH1	Zm00001eb004420	1:12250196–12,255,463	6	658	71472.1	8.38	49.81	− 0.692	Nucleus
ZmYTH2	Zm00001eb039740	1:210310094–210,320,323	4	450	50190.58	6.93	45.31	− 0.563	Nucleus
ZmYTH3	Zm00001eb057150	1:282728950–282,733,326	5	659	71761.08	8.54	40.22	− 0.83	Nucleus
ZmYTH4	Zm00001eb067780	2:4563164–4,564,080	1	119	13412.17	5.54	54.34	− 0.314	Cytoplasm
ZmYTH5	Zm00001eb071290	2:11567050–11,571,807	6	552	61338.13	5.82	51.46	− 0.702	Nucleus
ZmYTH6	Zm00001eb117080	2:239954979–239,959,093	4	578	64175.77	7.24	33.51	− 0.826	Nucleus
ZmYTH7	Zm00001eb117310	2:240440917–240,443,872	1	224	25092.15	6.26	56.83	− 0.594	Cytoplasm
ZmYTH8	Zm00001eb156740	3:214354338–214,360,795	2	748	81823.7	8.14	47.7	− 0.643	Nucleus
ZmYTH9	Zm00001eb171010	4:28298813–28,303,227	2	594	65169.67	5.72	47.78	− 0.771	Nucleus
ZmYTH10	Zm00001eb174360	4:42048184–42,055,378	2	720	78506.14	6.65	43.14	− 0.526	Nucleus
ZmYTH11	Zm00001eb190100	4:171575277–171,583,246	1	254	27638.41	5.61	39.99	− 0.029	Nucleus
ZmYTH12	Zm00001eb224690	5:43943939–43,957,624	2	640	69977.97	6.36	55.03	− 0.891	Nucleus
ZmYTH13	Zm00001eb260100	6:6553642–6,560,118	3	688	75204.08	6.43	46.73	− 0.565	Nucleus
ZmYTH14	Zm00001eb273850	6:101013873–101,020,540	2	643	70102.08	6.32	57.18	− 0.894	Nucleus
ZmYTH15	Zm00001eb300970	7:8485647–8,490,066	1	637	70349.94	7.24	33.43	− 0.804	Nucleus
ZmYTH16	Zm00001eb338390	8:24991222–25,000,760	1	351	38734.07	5.81	42.13	− 0.537	Nucleus
ZmYTH17	Zm00001eb353860	8:128871065–128,875,568	3	723	81080.6	8.82	51.19	− 0.554	Nucleus
ZmYTH18	Zm00001eb360390	8:154951149–154,958,427	5	450	50114.51	9.34	43.61	− 0.596	Nucleus
ZmYTH19	Zm00001eb390780	9:119065889–119,068,256	1	289	32669.46	7.72	38.09	− 0.739	Chloroplast
ZmYTH20	Zm00001eb403260	9:159244745–159,250,613	5	676	72976.01	8	50.56	− 0.622	Nucleus
ZmYTH21	Zm00001eb403960	9:160660388–160,669,701	4	613	70080.96	7.63	44.54	− 0.843	Nucleus
ZmYTH22	Zm00001eb414030	10:67408711–67,413,202	2	592	64827.53	5.76	49.58	− 0.753	Nucleus

### Phylogenetic analysis of *ZmYTH* genes

**Fig. 1:**
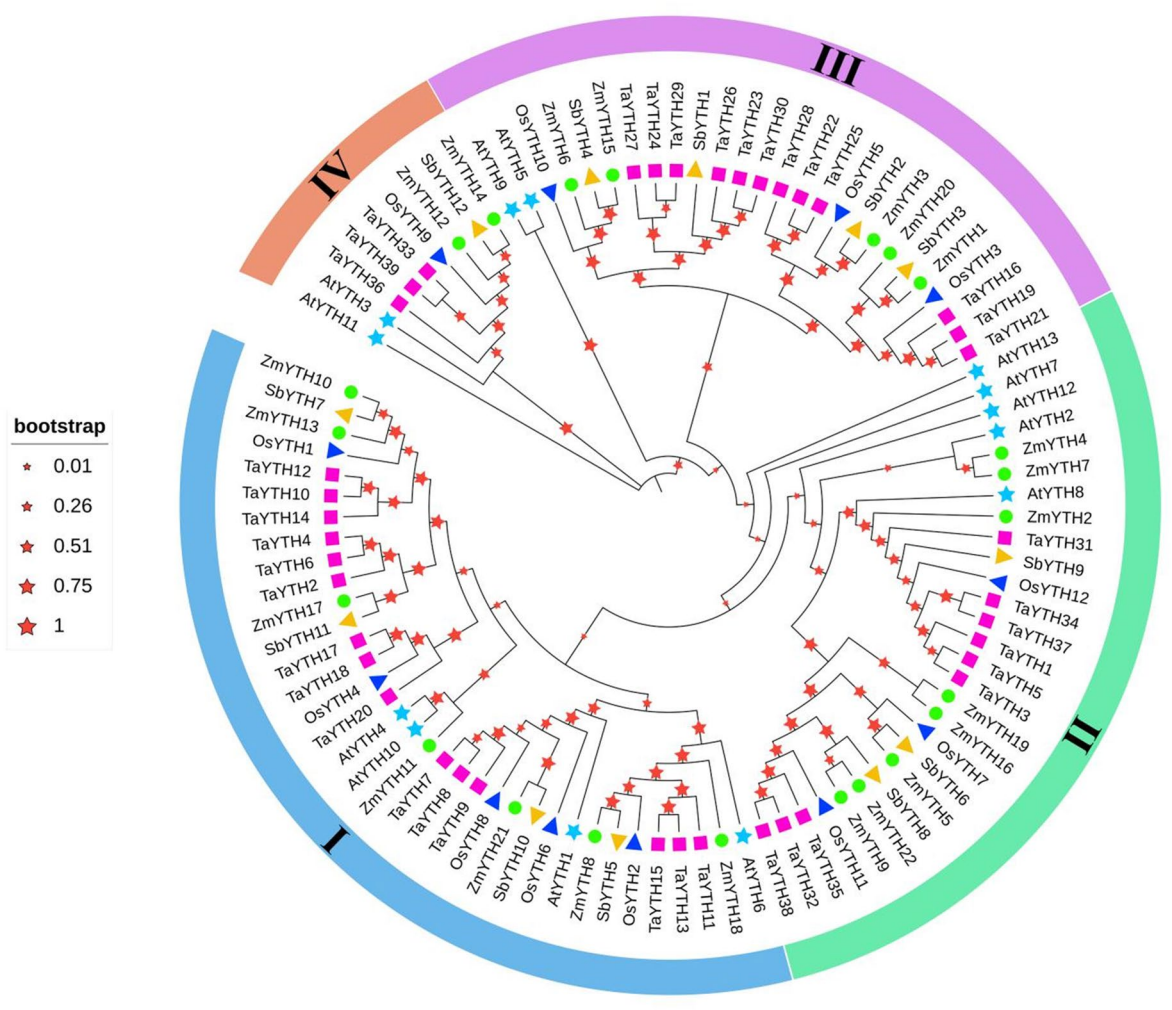
Phylogenetic analysis of YTH domain-containing proteins from maize and representative plant species. The phylogenetic tree was constructed using 22 ZmYTH proteins together with homologous YTH proteins from *Sorghum bicolor*, *Oryza sativa*, *Arabidopsis thaliana*, and *Triticum aestivum*. Multiple sequence alignment was performed using ClustalW, and the tree was generated using the Neighbor-Joining (NJ) method implemented in MEGA 11 with the JTT + I+G substitution model and 1,000 bootstrap replicates. The phylogenetic tree was visualized using the iTOL online tool. The YTH proteins were classified into four major clades (I–IV), indicated by different colored outer arcs. Different symbols represent proteins from different species: *Zm* (*Zea mays*), *Sb* (*Sorghum bicolor*), *Os* (*Oryza sativa*), *At* (*Arabidopsis thaliana*), and *Ta* (*Triticum aestivum*). Bootstrap support values are indicated by red stars at the nodes.

**Fig. 2:**
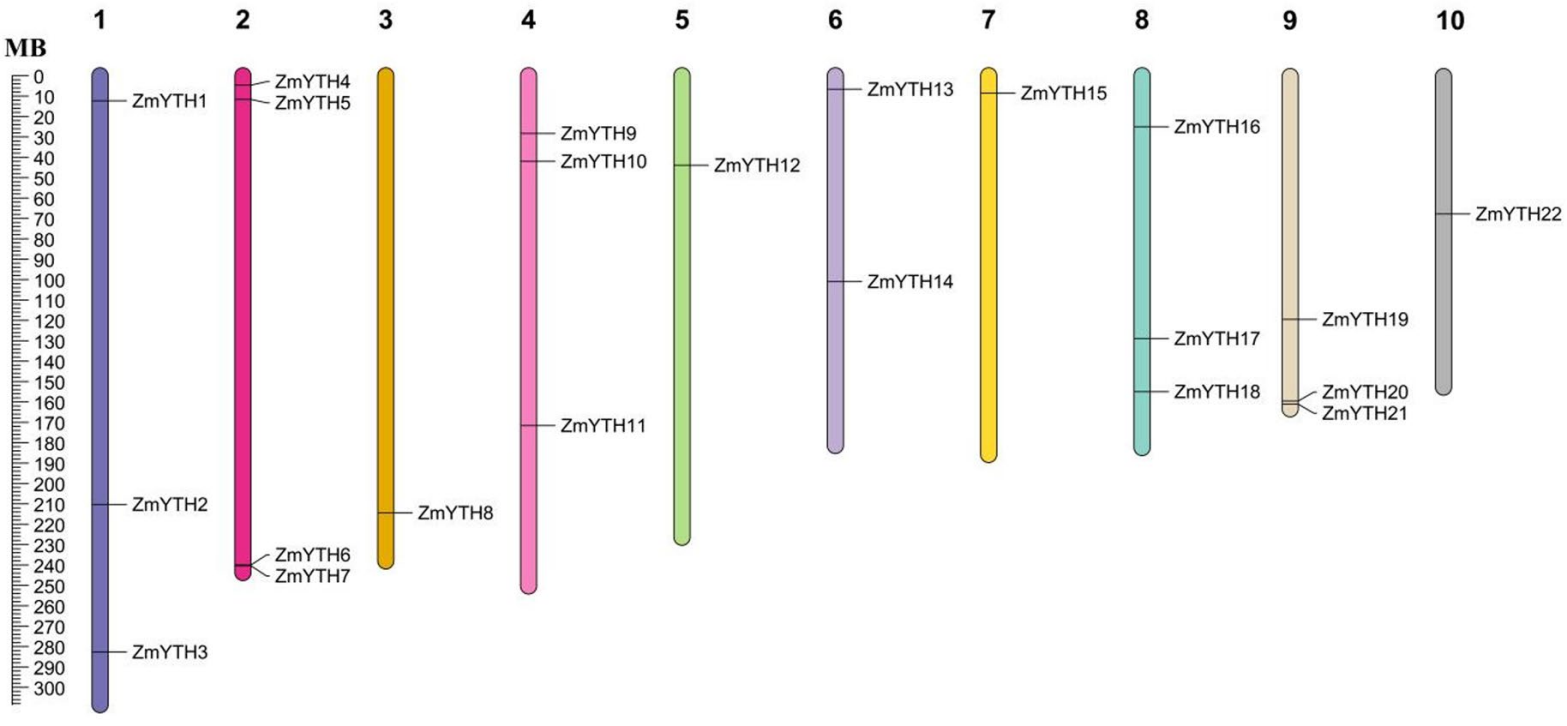
Chromosomal distribution of *ZmYTH* genes in maize. The physical positions of 22 *ZmYTH* genes on maize chromosomes (1–10) are shown according to the maize genome annotation. The vertical scale on the left represents chromosome length in megabases (Mb). Gene names are indicated at their respective chromosomal positions. Chromosomes 2 contain the highest number of *ZmYTH* genes.

An evolutionary relationship of maize YTH proteins was further analyzed by a phylogenetic tree based on 22 ZmYTH protein sequences and corresponding YTHs in *S. bicolor*, *O. sativa*, *A. thaliana*, and *T. aestivum*. The sequences were aligned through ClustalW and then NJ tree was generated in MEGA 11 under the JTT model with I + G, partial deletion, site coverage cutoff of 50%, and 1000 bootstrap replications. The phylogenetic tree was animated with iTOL (Fig. 1). In the phylogenetic tree, the YTH proteins were divided into four main clades, consistent with the previous classification of YTH proteins in other plant species^49^. YTH proteins from all five species were found in all the clades (Fig. 2). The clade I has the highest number of proteins (35), including 4 *At*, 4 *Sb*, 5 *Os*, 7 *Zm* and 15 *Ta*. In contrast, clade IV has the smallest number of proteins (9), including 2 *At*, 1 Sb, 1 *Os*, 2 *Zm*, and 3 *Ta*. Clades II and III contain 28 and 26 proteins, respectively. In this phylogenetic relation, most of ZmYTHs were present closer to the monocotyledon species. However, a few proteins, such as ZmYTH4, ZmYTH7 and ZmYTH11, showed relatively distant relationships from monocots and closer to Arabidopsis, suggesting functional differentiation. Phylogenetic analysis forms a natural base for functional prediction and evolutionary analysis of the maize YTH gene family.

### Chromosome localization, gene duplication, and Synteny relationships of *ZmYTH* genes

The *ZmYTH* genes are unevenly distributed among the ten maize chromosomes. Chromosome 1 (*ZmYTH1-ZmYTH3*), 4 (*ZmYTH9-ZmYTH11*), 8 (*ZmYTH16-ZmYTH18*), and 9 (*ZmYTH19-ZmYTH21*) each harbor three genes, while chromosome 2 contains the highest number, with four genes (*ZmYTH4–ZmYTH7*). Chromosome 6 contains two genes (*ZmYTH13* and *ZmYTH14*), while chromosome 3 (*ZmYTH8*), 5 (*ZmYTH12*), 7 (*ZmYTH15*), and 10 (*ZmYTH22*) each contain one gene. These findings confirm the uneven distribution of YTH genes across the maize chromosomes.

**Fig. 3:**
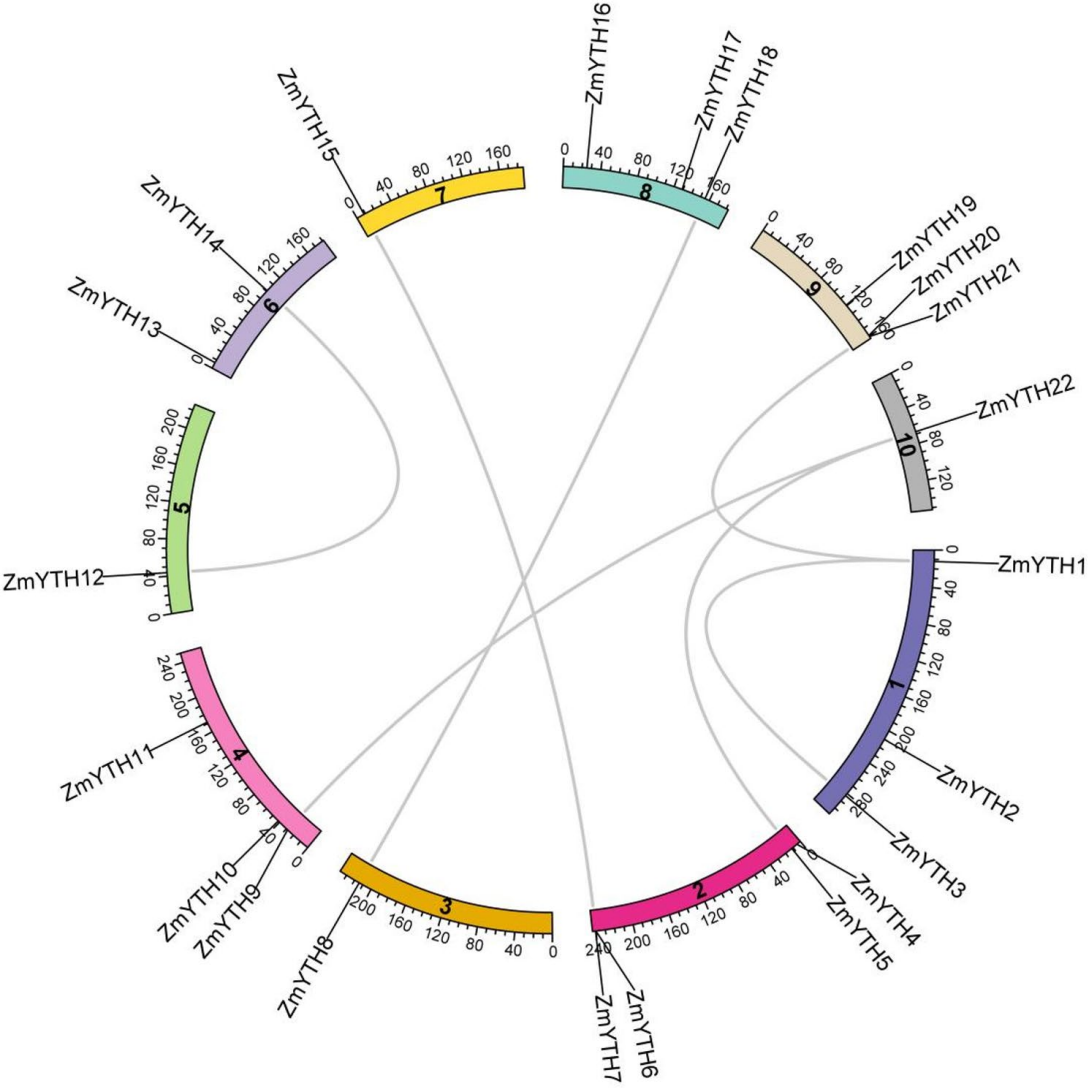
Intragenomic synteny of *ZmYTH* genes in maize. The circular diagram represents the positions of *ZmYTH* genes on the 10 maize chromosomes (with Mb scale).

**Fig. 4:**
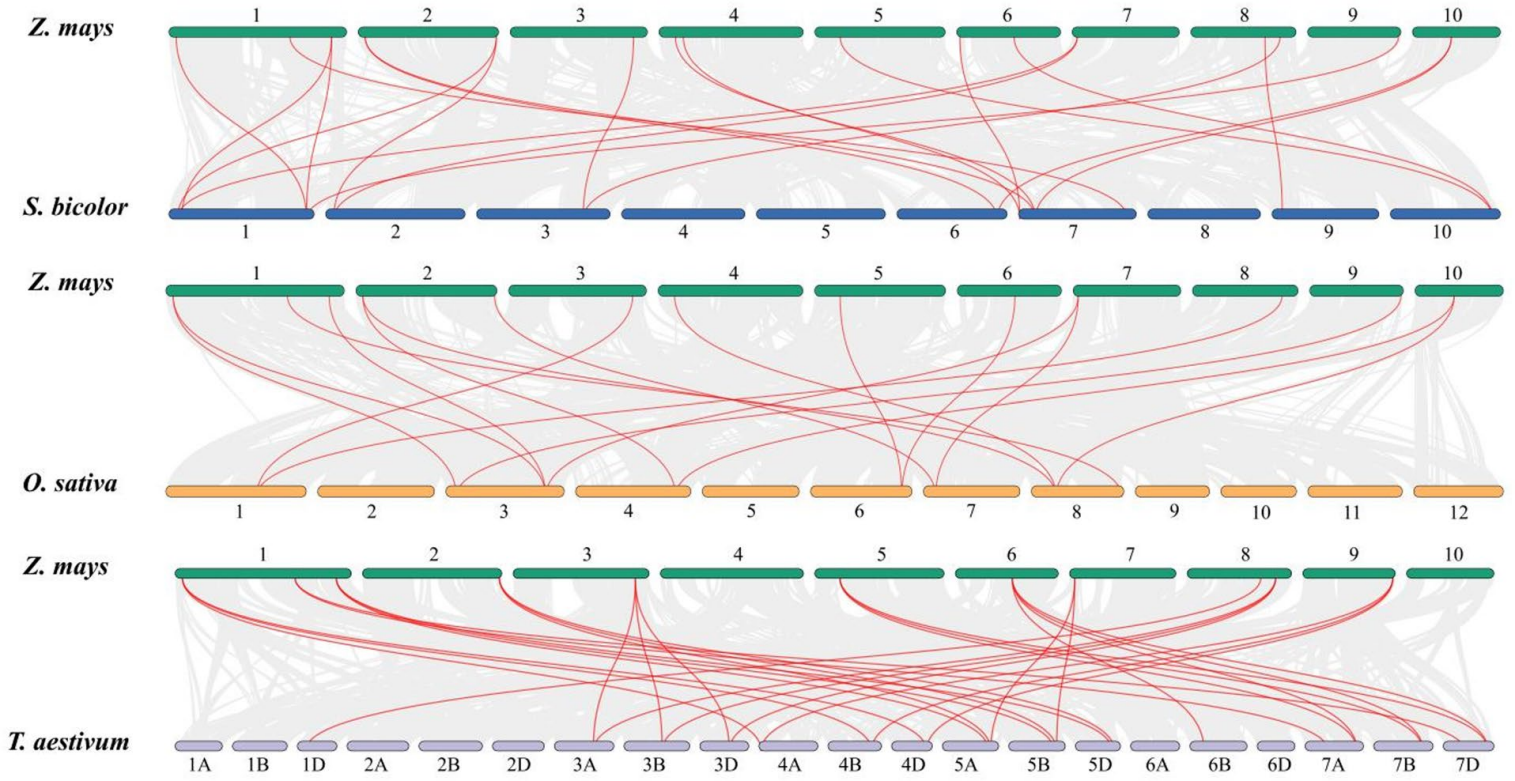
Comparative synteny analysis of *ZmYTH* genes between *Zea mays*, *Sorghum bicolor*, *Oryza sativa*, and *Triticum aestivum*. Red lines indicate syntenic relationships between orthologous YTH gene pairs, while the grey lines represent genome-wide collinearity. The conserved syntenic blocks highlight the evolutionary stability of ZmYTH genes across monocot species.

Furthermore, we conducted the duplication and synteny analysis. W**e** found the 7 gene duplication events in maize YTH gene family through segmental duplication (SD). These duplicated genes were distributed across all chromosomes (Fig. 3). The results demonstrated that segmental duplication was the primary driver of the architecture of the *ZmYTH* gene family. To investigate the evolutionary conservation of *ZmYTH* genes across plant species, a comparative synteny analysis was conducted between maize and three other plant species (*S. bicolor*, *T. aestivum*, and *O. sativa*) (Fig. 4; Supplementary Table S3). There were 16, 13, and 11 *ZmYTHs* that had collinearity with the YTHs of *S. bicolor*, *O. sativa*, and *T. aestivum*, respectively. Notably, 10 *ZmYTH* genes (*ZmYTH1*, *ZmYTH2*, *ZmYTH3*, *ZmYTH6*, *ZmYTH8*, *ZmYTH12*, *ZmYTH14*, *ZmYTH15*, *ZmYTH18*, and *ZmYTH20*) were showed conserved collinearity across all three species.

### Gene structure and conserved motifs of *ZmYTHs*

**Fig. 5:**
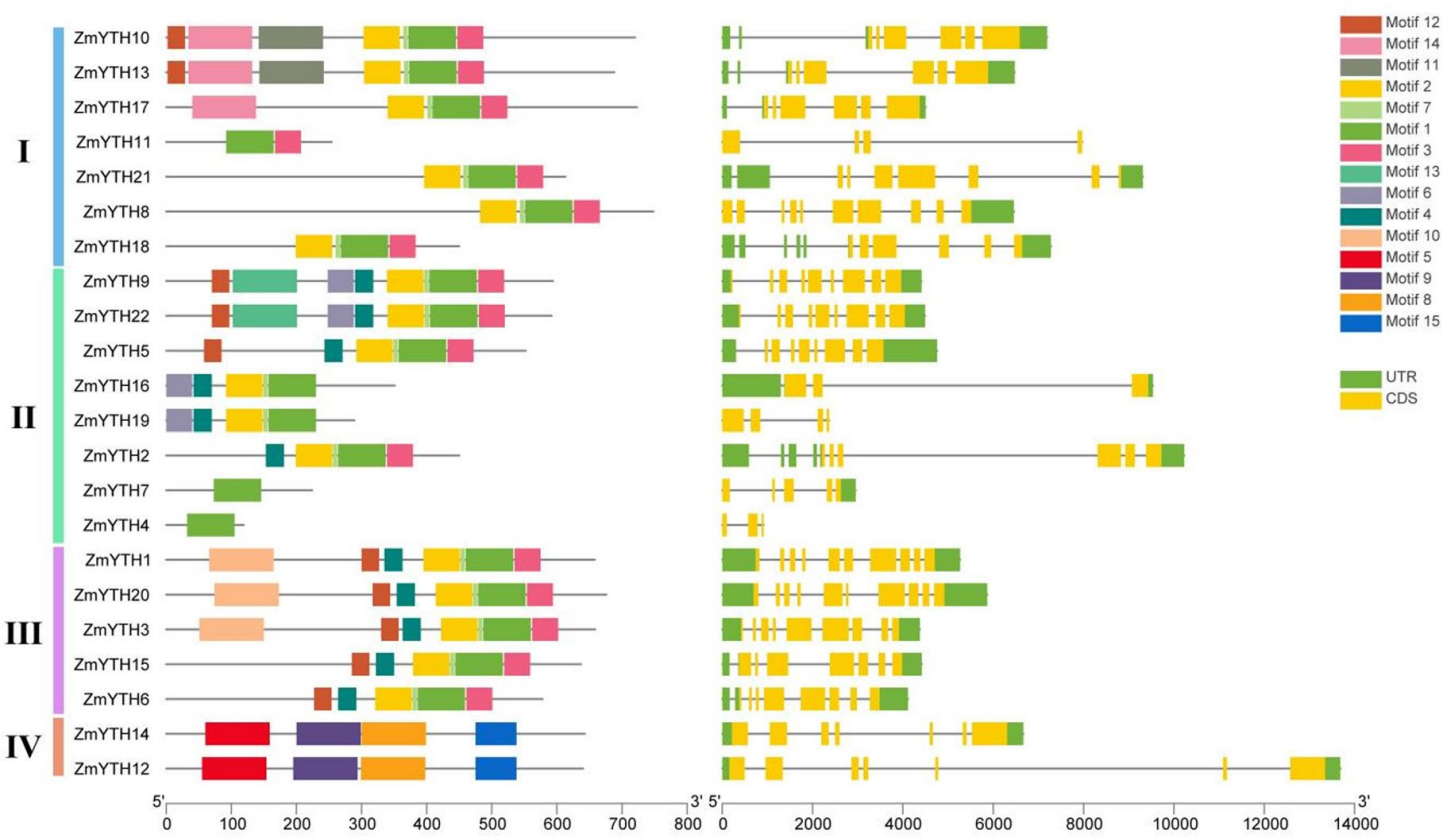
Gene structure and conserved motifs of ZmYTH proteins. The left panel shows the distribution of 15 conserved motifs, identified by MEME, across the ZmYTH proteins, color-coded and grouped by phylogenetic clades I–IV. The right panel illustrates the exon-intron organization, with coding sequences (CDS) in yellow and untranslated regions (UTRs) in green. The motif analysis reveals that conserved motifs, such as Motifs 1, 3, and 7, are predominantly located in the most of ZmYTHs, whereas other motifs exhibit varying distributions across individual genes, reflecting both functional conservation and clade-specific divergence. The gene structures were visualized using TBtools.

To further elucidate structural characters and diversity of the *ZmYTH* gene family, we assessed its gene structure and conserved motifs. The exon-intron structure and conserved motif locations of the *ZmYTH* genes are depicted in Fig. 5. The exon-intron structure of *ZmYTH* genes showed diversity. The number of exons ranges from 3 to 10 among the *ZmYTH* members. For example, *ZmYTH1*, *ZmYTH8* and *ZmYTH20* each contain 10 exons, whereas *ZmYTH4* and *ZmYTH16* contain only 3 exons. Interestingly, most of *ZmYTHs* genes contain untranslated regions (UTRs) at both 5’ and 3’ ends, except three genes (*ZmYTH4*, *ZmYTH11*, and *ZmYTH19*) which do not have any UTRs, while *ZmYTH8* contain only one UTR at 3’ end. All UTRs containing genes have one UTR at 3’ end, while it varies from 1–5 at 5’ end (Fig. 5). This diversity of organization indicates that the *ZmYTH* genes may have different functions in gene regulation in maize.

Motifs of the ZmYTH proteins were analyzed by MEME, and their distribution is displayed in Fig. 5. In total, 15 different motifs were identified across the gene family, and Motif 1, Motif 7, and Motif 3 were among the most common. These motifs are indispensable for the RNA-binding activity of the YTH domain, a characteristic essential to ZmYTH proteins. Furthermore, we found that some motifs were specific to the specific groups, such as motif 14, which was present only in the 3 members (ZmYTH10, ZmYTH13, ZmYTH17) of Group I, whereas motif 6 was only present in the 4 members (ZmYTH9, ZmYTH16, ZmYTH19, ZmYTH22) of Group II. Besides that, motifs 5, 8, 9 and 15 were only present in the Group IV members. These results suggested that these conserved motifs may play vital roles in subgroupspecific function.

### Promoter analysis of *ZmYTHs*

**Fig. 6:**
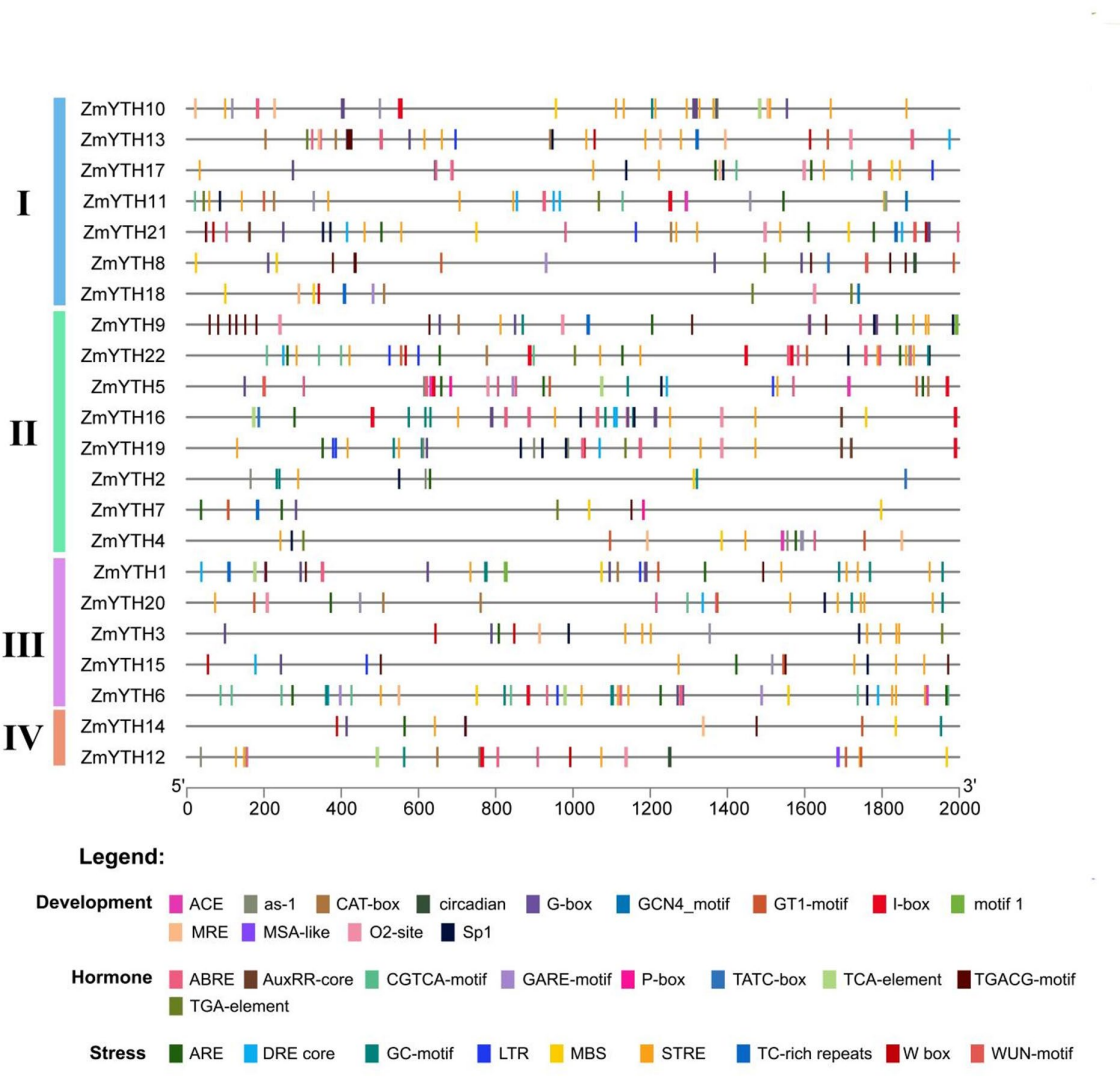
Promoter analysis of *ZmYTH* genes. The 2000-bp promoter regions upstream of the transcription start sites of *ZmYTH* genes were analyzed for cis-acting elements. The identified cis-regulatory elements were presented into three categories, including development, hormone and stress related.

We performed promoter analysis to identify the cis-regulatory elements of the *ZmYTH* genes. We presented the identified various cis-regulatory elements broadly into three categories, including, development, hormone and stress related (Fig. 6 and Table S4). We obtained 13 development-related cis-elements (e.g., ACE, as-1, CAT-box, circadian, G-box) that might be involved in the regulation of various developmental genes that control processes such as seed growth and initial plant growth. Furthermore, 9 hormone-responsive cis-elements were identified, (e.g., ABRE, AuxRR-core, CGTCA-motif, GARE-motif, P-box), which suggested that *ZmYTH* genes can help regulate hormone signaling pathways, especially related to ABA, auxins, and jasmonic acid. In addition, stress-responsive cis-elements (e.g., ARE, DRE-core, GC-motif, MBS) were found, and it suggests that *ZmYTH* genes might be participating in the plant’s response to stresses. The cis-elements are distributed differently in the *ZmYTH* gene family. Some members contain more development and hormone related elements, while some have more stress related elements. This pattern of unequal distribution of cis-elements across the gene family implies that *ZmYTH* genes are also functionally specialized, with some associated with developmental processes and others with stress adaptation processes. Such outcomes indicate the complexity of the *ZmYTH* family of genes, which might be used for developmental and stress tolerance control in maize.

### Protein-protein interaction (PPI) network and miRNA target prediction for *ZmYTH* genes

**Fig. 7:**
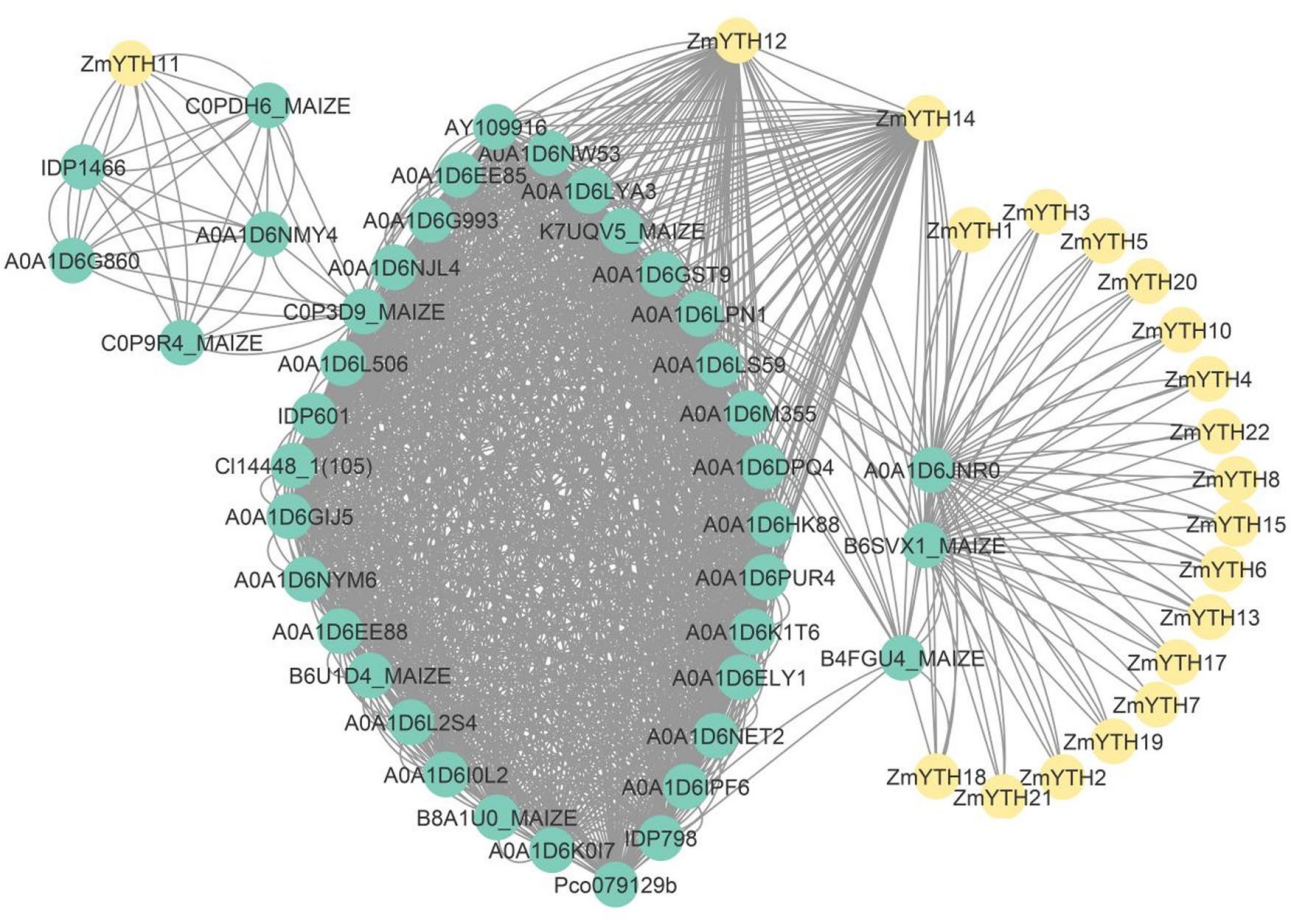
PPI network of *ZmYTH* genes in maize. The network, constructed using the STRING database, shows central interactions between ZmYTH proteins (Groups I and IV) and maize proteins involved in signal transduction, stress responses, and metabolism, highlighting their pivotal roles in regulatory networks.

To examine the functional relationships and regulatory roles of *ZmYTH* genes, we conducted Protein-Protein Interaction (PPI) Network Analysis and miRNA target prediction. These studies offer detailed insights into how *ZmYTH* genes may be involved in cellular functions and in their post-transcriptional regulation. The PPI network constructed from the STRING database showed clear interactions among ZmYTH proteins and other maize proteins, indicating that these genes are important to multiple biological processes (Table S5). *ZmYTH* genes such as *ZmYTH12* and *ZmYTH14* were relatively core in the network to associate proteins implicated in signal transduction, and metabolic regulation, as shown in Fig. 7. The network showed that some *ZmYTH* genes were central hub nodes; these genes may play important roles in the regulatory networks of maize.

**Fig. 8:**
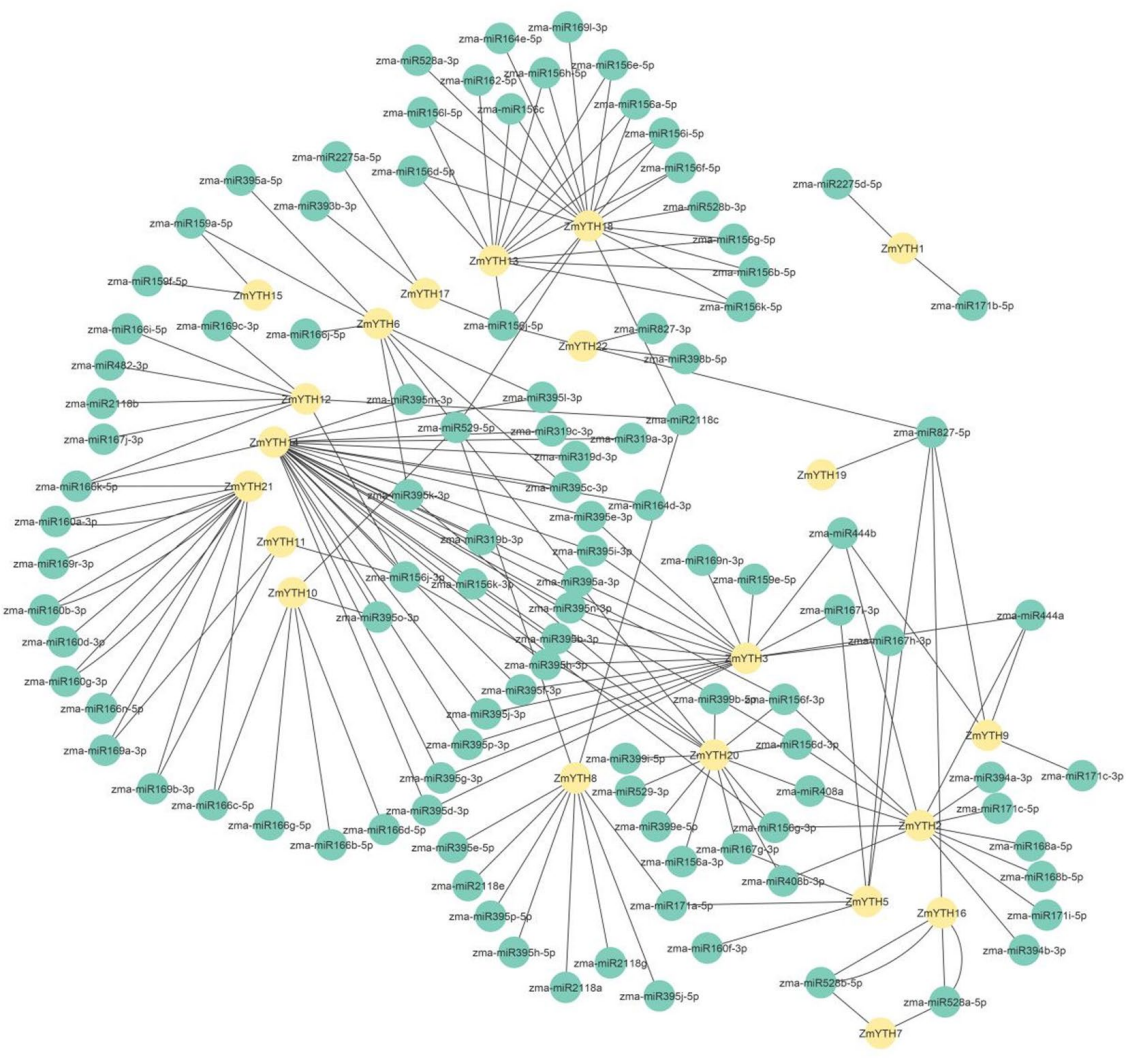
miRNA-target interaction network of *ZmYTH* genes in maize. The network shows the interactions between *ZmYTH* genes (yellow) and maize miRNAs (green). Multiple miRNAs, including zma-miR156, zma-miR164, and zma-miR167, target several *ZmYTH* genes, indicating a complex layer of post-transcriptional regulation. The network highlights the involvement of *ZmYTH* genes in regulating developmental and stress response pathways in maize.

Meanwhile, miRNA target prediction revealed that several maize miRNAs, particularly members of the zma-miR156, zma-miR167, and zma-miR395 families (including various isoforms such as 3p and 5p variants), targeted 21 *ZmYTH* genes, whereas ZmYTH4 was not predicted to be targeted by any miRNA. Among the target ZmYTHs mostly targeted by multiple miRNAs, implying the complex post-transcriptional level regulation of genes (Fig. 8, Table S6). Notably, ZmYTH14 was targeted by the highest number of miRNAs (24). Based on the miRNA-target interaction network, *ZmYTH* genes play diverse roles in complex regulatory networks, regulating developmental and stress-response processes in maize. Overall, the integration of PPI network analysis with miRNA target prediction provided a system-level view of *ZmYTH* gene regulation. The results suggest that both protein–protein interactions and miRNA-mediated pathways may contribute to the regulation of *ZmYTH* gene functions, potentially influencing maize growth, development, and stress responses.

### Expression profiles of *ZmYTHs* in various tissues and under different stresses

**Fig. 9:**
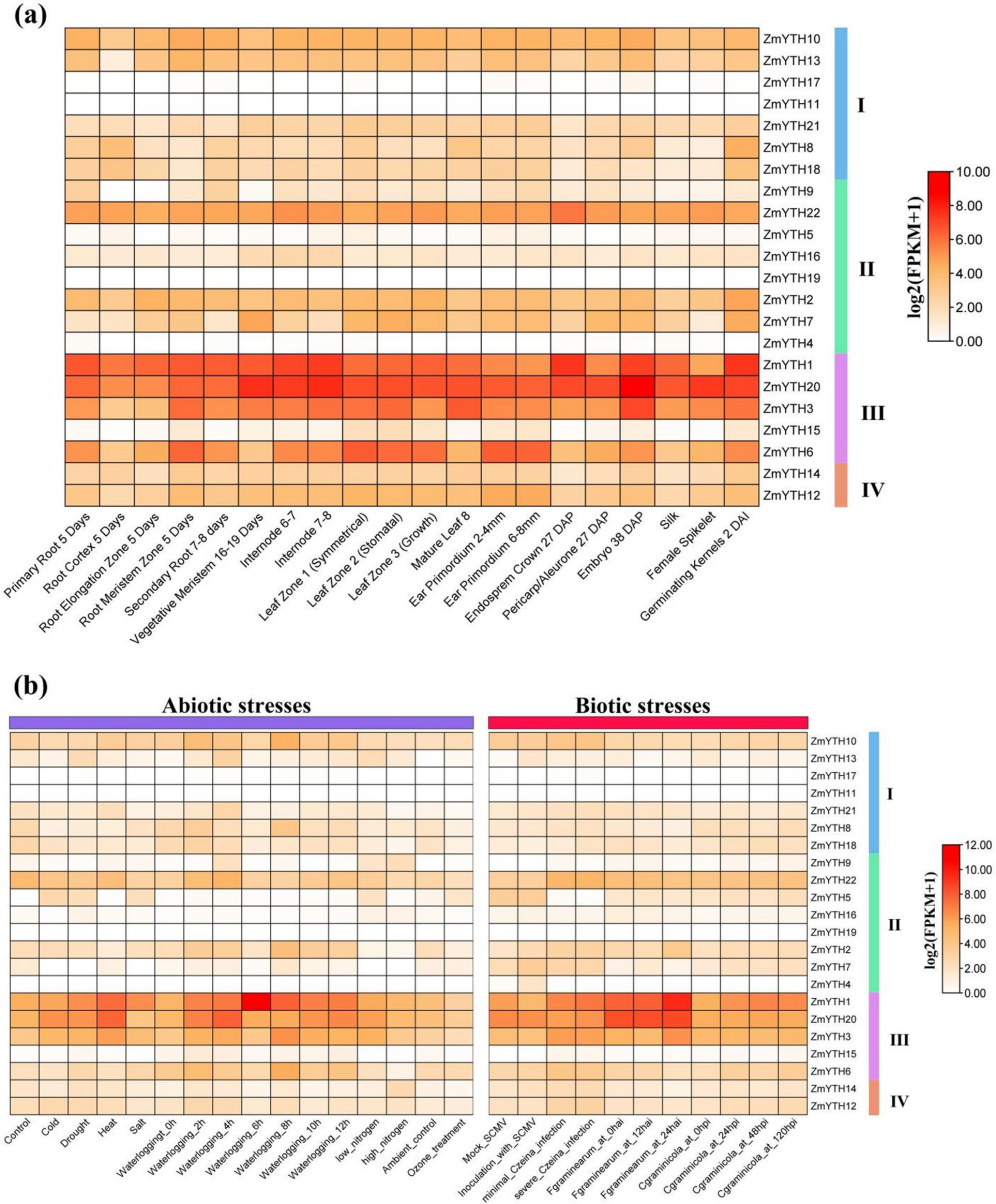
Expression profiles of *ZmYTH* genes in various tissues at various developmental stages and under various biotic and abiotic stresses. (**A**) Expression patterns of *ZmYTHs* in different tissues at different developmental stages. (**B**) Expression profiles of the *ZmYTHs* in response to abiotic and biotic stresses. The expression profiles of each *ZmYTH* were presented as log2(FPKM + 1) values. The color bar represents expression value intensity.

The expression patterns of the *ZmYTH* genes were studied in various tissues and in response to different stress treatments to assess their roles during development, as shown in Figs. 9A and B and Tables S7, S8. These findings revealed distinct patterns in the spatial and temporal expression of *ZmYTH* genes, indicating their roles in regulating developmental processes and responding to environmental stress.

The expression patterns of *ZmYTH* genes exhibited significant differences across tissues, whereas some genes, such as *ZmYTH1*, *ZmYTH3*, *ZmYTH6*, *ZmYTH20*,* and ZmYTH22* showed high expression levels in all tissues, indicating their role from the overall plant development to specific tissues development. essential roles for these genes, particularly in early plant development, root elongation, and seed formation. On the other hand, *ZmYTH15* showed relatively higher expression in leaf, ear primordium and germinating kernels, indicating their potential involvement in leaf development and differentiation and ear development (Fig. 9A, Table S7). These results imply that *ZmYTH* genes are functionally divergent and have distinct functions at various stages of maize development.

Under abiotic stresses such as drought, heat, and salt stress, the expression of *ZmYTH* genes exhibited variations, suggesting possible involvement in stress responses (Fig. 9B and Table S8). One such gene is *ZmYTH1*, which was significantly upregulated under drought, cold, salt, heat and waterlogging stresses, while another follow-up homologous gene of *ZmYTH3* in maize also showed significant upregulation. The genes are likely associated with early, durable responses to stress, contributing to the plant’s ability to recover from such stress. In contrast, *ZmYTH7* and *ZmYTH22* exhibited downregulation under drought and salt stresses.

The expression profiles of *ZmYTH* genes were also analyzed under biotic stress, including fungal infection by *Fusarium graminearum* and *Cochliobolus carbonum*. Notably, *ZmYTH1* and *ZmYTH20* were strongly upregulated, particularly at 12–24 h post-inoculation, suggesting their potential involvement in maize defense against fungal pathogens (Fig. 9B; Table S8). These findings indicate that these genes may contribute to biotic stress response and defense-related signaling pathways.

Conclusively, the tissue-specific and stress-responsive expression patterns of *ZmYTHs* demonstrate that they are dynamically regulated and likely perform diverse functions in maize. These expression profiles indicate that these genes play a role in both development and stress responses, with certain members predominantly associated with growth regulation, while others primarily regulate stress resistance or immune responses. Such functional divergence suggests a possible role for *ZmYTH* genes in stress responses and in ensuring that maize can grow and survive under different stressful environmental conditions.

### qRT-PCR validation of *ZmYTH* genes under drought, salt, and heat stresses

**Fig. 10:**
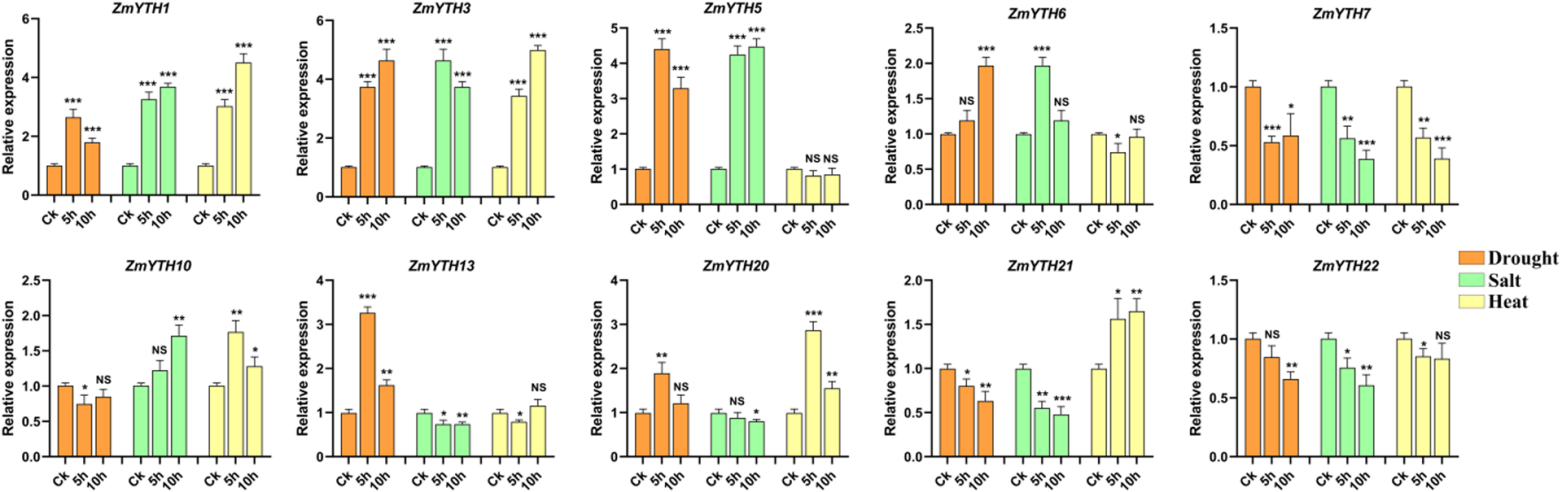
qRT-PCR validation of *ZmYTH *gene expression under drought, salt, and heat stresses. Drought stress was induced using 20% PEG 6000 in Hoagland’s solution, salt stress using 150 mM NaCl, and heat stress at 38 °C. Leaf samples were collected at 0 h (control), 5 h, and 10 h after treatment. Relative expression levels of genes at 5 and 10 h are shown, with statistical significance marked (*p < 0.05, **p < 0.01, ***p < 0.001).

The expression of *ZmYTH* genes was verified under drought, salt, and heat stresses using qRT-PCR. Figure 10 shows the differences in expression levels of *ZmYTH* genes at 5 and 10 h after treatment under these stress conditions. Only a subset of *ZmYTH* genes exhibited consistent expression patterns across all stress conditions. Notably, *ZmYTH1* and *ZmYTH3* were strongly upregulated under all stresses at both time points, whereas *ZmYTH7* and *ZmYTH22* were consistently downregulated across all treatments. *ZmYTH5* showed significant upregulation under drought and salt stresses. Similarly, *ZmYTH6* was significantly upregulated under drought and salt conditions at different time points but exhibited downregulation under heat stress. *ZmYTH10* was downregulated at 5 h under drought stress but upregulated under salt and heat stresses. In contrast, *ZmYTH13* was upregulated under drought stress but downregulated under salt and heat conditions. *ZmYTH20* displayed upregulation under drought and heat stresses, while being downregulated under salt stress at 10 h. Finally, *ZmYTH21* was downregulated under drought and salt stresses but showed clear upregulation under heat stress.

Overall, the qRT-PCR data revealed that the expression patterns of *ZmYTH* genes vary with responses to drought, salt, and heat stress. Some genes (notably *ZmYTH1* and *ZmYTH3*) were highly up-regulated and more pronounced at early time points of stress, suggesting they may play a crucial role in adaptation to stress. The apparent changes in other genes (*ZmYTH6* and *ZmYTH22*) were less responsive, suggesting that they serve a separate purpose in stress management or, later on, are involved in other biological processes.

## Discussion

RNA modifications, particularly N6-methyladenosine (m^6^A), play central roles in post-transcriptional gene regulation in plants by influencing RNA stability, splicing, and translation during growth, development, and stress responses. YTH domain-containing proteins act as key m^6^A readers that mediate downstream regulatory processes. Although YTH proteins have been extensively characterized in model plants such as Arabidopsis and rice, their genomic organization and functional roles in maize remain poorly understood. The present findings provide new insights into the *ZmYTH* gene family, highlighting their evolutionary expansion, structural diversity, and potential roles in maize development and stress adaptation.

### Evolutionary expansion and structural diversification of *ZmYTH* genes

The *ZmYTH* gene family in maize exhibits clear evidence of evolutionary expansion and structural diversification, suggesting its potential importance in RNA metabolism and post-transcriptional gene regulation. A total of 22 YTH domain-containing proteins were identified, providing a basis for exploring their roles in m^6^A-mediated regulation of RNA stability, splicing, and translation during plant growth, development, and stress responses^17,50^.

The uneven chromosomal distribution of *ZmYTH* genes, with the highest density observed on chromosomes 2, suggests that gene duplication events and chromosomal rearrangements may have contributed to the expansion of this gene family in the maize genome. Similar nonuniform distribution patterns have been reported in other plant species, such as *Gossypium* and *Cucumis sativus*, where they are associated with evolutionary adaptation and functional divergence^51,52^. In addition, subcellular localization predictions indicate that most ZmYTH proteins are localized in the nucleus, consistent with their established roles in post-transcriptional gene regulation in *A. thaliana*^49^, although experimental validation is required. Phylogenetic analysis further demonstrated that ZmYTH proteins are grouped into four major clades, consistent with classifications reported in *A. thaliana* and *O. sativa*^25,49^. The nomenclature of ZmYTH1–ZmYTH22 follows the appearance of them at the chromosomes. The clustering of ZmYTH proteins with homologs from monocot species supports their evolutionary conservation and suggests potential conserved roles in m^6^A-mediated RNA regulation. In contrast, the phylogenetic separation from dicot YTH proteins reflects lineage-specific divergence, which may contribute to functional specialization^53^. Moreover, gene duplication events, including segmental and whole-genome duplications, appear to have played a significant role in the expansion of the YTH gene family, as also observed in wheat and rice^31,49^.

Structural analysis revealed considerable variation in protein length (119–748 amino acids) and physicochemical properties among *ZmYTH* members, indicating potential functional diversification. Similar variability has been reported in other plant species, including *Gossypium spp.*, where differences in protein length and splicing patterns have been observed^52,54,55^. Such variation is often associated with alternative splicing and adaptive evolution in RNA-binding protein families. The negative GRAVY values further indicate that ZmYTH proteins are hydrophilic, which is consistent with their ability to interact with RNA and other macromolecules in the cellular environment^56^. Furthermore, synteny analysis revealed conserved genomic relationships among YTH genes in maize, rice, and sorghum, suggesting that these genes originated from a common ancestral locus prior to the divergence of grass species^49^. However, the absence of certain orthologs, such as *ZmYTH11* and *ZmYTH16*, in related species indicates possible lineage-specific gene loss or functional divergence^25,53^. Collectively, these findings suggest that the *ZmYTH* gene family has evolved through a combination of duplication, conservation, and diversification.

### Gene architecture, conversion, and functional divergence

The gene architecture of *ZmYTH* genes provides important insights into their evolutionary conservation and functional divergence. The exon–intron organization of these genes is generally conserved within the same phylogenetic subgroups, suggesting that they originated from common ancestral genes and have retained essential functional roles. Similar patterns of conserved gene structure have also been reported in YTH gene families of other plant species, including barley and rice^11,49^. Despite this conservation, variations in exon number and gene length among subgroups indicate structural divergence that may contribute to functional specialization following gene duplication. Gene duplication appears to have played a critical role in the expansion of the *ZmYTH* family, as evidenced by paralogous gene pairs such as *ZmYTH8–ZmYTH18* and *ZmYTH6–ZmYTH15*, which exhibit strong collinearity within the maize genome^51,57^. At the same time, duplication events, particularly segmental and whole-genome duplications, may have facilitated functional diversification within the gene family^58,59^.

Further support for functional conservation and divergence is provided by the analysis of conserved motifs among ZmYTH proteins. Members within the same phylogenetic subgroup generally share similar motif compositions, indicating conserved functional roles. In contrast, variations in motif distribution among different subgroups suggest divergence in regulatory functions, reflecting evolutionary adaptation within the YTH gene family^11^. Collectively, these findings highlight a balance between structural conservation and functional diversification, enabling *ZmYTH* genes to maintain core regulatory roles while adapting to specific biological processes in maize^60^.

### Regulatory networks and expression dynamics of *ZmYTH* genes

The functional roles of *ZmYTH* genes are further supported by their involvement in complex regulatory networks and dynamic expression patterns. PPI network analysis revealed that ZmYTH proteins are extensively interconnected with diverse maize proteins, particularly those involved in signal transduction and metabolic pathways, suggesting their potential roles in regulatory processes. In addition, miRNA target prediction analysis identified several miRNAs, particularly members of the zma-miR156 and zma-miR395 families (including multiple 3p/5p isoforms), that potentially regulate *ZmYTH* genes, suggesting an additional layer of post-transcriptional control^61,62^.

YTH domain-containing proteins function as m6A readers in plants, mediating post-transcriptional regulation by recognizing methylated RNA and influencing RNA stability, splicing, and translation^23,63^. Consistent with these regulatory roles, expression profiling revealed that *ZmYTH* genes exhibit distinct tissue-specific and developmental stage-specific expression patterns in maize. For instance, *ZmYTH1*, *ZmYTH3*, *ZmYTH6*, *ZmYTH20*, and *ZmYTH22* showed high expression levels in all tissues, indicating their role from the overall plant development to specific tissues development. On the other hand, *ZmYTH15* showed relatively higher expression in leaf, ear primordium and germinating kernels, indicating their potential involvement in leaf development and differentiation and ear development^64^. These differential expression patterns support the functional specialization of *ZmYTH* genes and highlight their diverse roles in maize biological processes.

### Roles in stress responses and future perspectives

The stress-responsive expression patterns of *ZmYTH* genes highlight their potential roles in maize adaptation to environmental challenges. Several *ZmYTH* genes, including *ZmYTH1* and *ZmYTH3*, exhibit significant upregulation in response to abiotic stresses such as drought, heat, and salinity, suggesting their involvement in regulating stress-responsive pathways. These observations are consistent with previous studies demonstrating that YTH domain-containing proteins play important roles in plant stress responses through m^6^A-mediated post-transcriptional regulation^49,51,53^. In addition to abiotic stress, the increased expression of *ZmYTH* genes in response to fungal infection further indicates their potential involvement in biotic stress defense mechanisms in maize^45,65^. The ability of *ZmYTH* genes to respond to both abiotic and biotic stresses suggests that they may function as central regulators in coordinating plant responses to diverse environmental conditions. The qRT-PCR validation results further support the reliability of the observed expression patterns, particularly for *ZmYTH1* and *ZmYTH3* under stress treatments, confirming their potential functional roles in stress adaptation^66^. These findings reinforce the importance of *ZmYTH* genes in maize growth and stress resilience.

Overall, the results presented in this study provide important insights into the *ZmYTH* gene family as regulators of post-transcriptional gene expression. Given the growing role of m^6^A modifications in plant stress adaptation^56,66^, further functional studies, including gene knockout and overexpression analyses, will be important to elucidate the precise biological roles of *ZmYTH* genes. Such studies may contribute to the development of stress-resilient maize varieties and improved crop productivity under adverse environmental conditions.

## Conclusion

The phylogeny and diversity of structural characteristics of 22 YTH domain-containing RNA-binding proteins (*ZmYTHs*) were analyzed, and these genes showed an uneven distribution across chromosomes. Phylogenetic analysis classified the *ZmYTHs* into four main clades, indicating that YTH genes were relatively conserved yet functionally diverse during evolution. Gene duplications have contributed to the expansion of the *ZmYTH* gene family, potentially increasing its regulatory potential for maize adaptability to stress. Expression profiling and qRT-PCR validation results show that *ZmYTHs*, especially the cluster comprising *ZmYTH1* and *ZmYTH3* are strongly upregulated by drought, salt, and heat stresses, whereas *ZmYTH7* significantly downregulated, suggesting their involvement in early stress responses. The results of this study suggest that ZmYTH proteins may play an important role in regulating RNA metabolism under abiotic stress. In sum, the *ZmYTH* gene family plays an important role in regulating RNA, especially under abiotic stress in maize. More functional studies are also required to clarify these genes at the molecular level, which could be used to breed crops with enhanced resistance to stress.

## Supplementary Information

Below is the link to the electronic supplementary material.

Supplementary Material 1

## Data Availability

All data generated or analyzed during this study are included in this published article.
